# Supplementary material for: Study of SarA by DNA Affinity Capture Assay (DACA) Employing Three Promoters of Key Virulence and Resistance Genes in Methicillin-Resistant Staphylococcus aureus
Source: Antibiotics (Basel). 2022 Nov 28;11(12):1714. doi: 10.3390/antibiotics11121714 (PMC9774152; doi:10.3390/antibiotics11121714)
Supplement: Supplementary file 1 [file antibiotics-11-01714-s001.zip › antibiotics-2011834-supplementary.pdf]

Supplementary data

Supplementary Table S1. Primer sequences used in this study

Primer name	Sequence (5' → 3')
mecA-F	Biotin-TCTCACTAAAAAAATTACACATA
mecA-R	CAATATACTCCTTATATAAGACTAC
sarA-F	Biotin-ATTAACTTTTAGCTTATCATTTTAACTTG
sarA-R	GTTTAAAACCTCCCTATTTGAT
sarR-F	Biotin-TGTCAATGTATGTAGGTTGATAAGAAGAG
sarR-R	TATTAAACCACTCCTCTGATGCA

Supplementary Table S2. PCR product sequence

Upstream region PCR product	Size (bp)	Sequence (5' → 3')
mecA	171	TCTCACTAAAAAAATTACACATATCGTGAGCAATGAACT GATTATACTTAACATTAAAAAAGATGATAACACCTTCTA CACCTCCATATCACAAAAATTATAACATTATTTTGACATA AATACTACATTTGTAATATACTACAAATGTAGTCTTATAT AAGGAGTATATTG
sarA	947	ATTAACTTTTAGCTTATCATTTTAACTTGTAATTATTTTTA AAAGTGATAAGCTATTTTTTTGTGGTCTAAAAATCTTTAG AAAAGCGTTGATTTGGGTAGTATGCTTTGACACAACAAA TTTTAATTTAGCAAATTCGATAGTCAACTCATTCTTAAGA CCTAAATTAATGTTATTTTTTAATAATTTACACCAAATTA ATAGCAAAAATTATGTTATTCGTGCTAATATTTCATAGTT GGTTATTCAATTAATTAAAAATAAGTCAAAATGCACAAC TTTTTATAATTCATTGAGTCGAGTTTGAAAAATAAAAGTG CTTTAATGCATGATCAATTATCGTACTTTCTATTATTTGTT ACCCGTTATCAATCGGAATAACGTATAGACACTTTAACG TGCTATAGATTGGTTTTAATCACTAAATTAATGTGTTTTT CTTATCATTAAAACTGCACTGAGAATTACTAAATTAAAA AAATTATAAAAATTTTTCATTTTTAGTGATAAAATTCTGA AAAATGGGTATAAATAGTAGAAGAAGTTAACTTGGAAG AGTTAAGCTATAACAAAGAATCTCTTTAGACACACATTG AATATCGAAACATTTAATTGCGCTAAATCGTTTCATTAAA TAAATTACCTTGTATTGTCGATTAAATTAAGGTAAATTAT AAAAAATGCTGATATTTTTGACTAAACCAAATGCTAACC CAGAAATACAATCACTGTGTCTAATGAATAATTTGTTTTA TAAACACTTTTTTGTTTACTTCTCATTTTTAATTAGTTATA ATTAACTAAATAATAGAGCATTAAATATATTTAATAAAA CTTATTTAATGCAAAATTATGACTAACATATCTATAATAA ATAAAGATTAGATATCAATATATTATCGGGCAAATGTAT CGAGCAAGATGCATCAAATAGGGAGGTTTTAAAC
sarR	427	TGTCAATGTATGTAGGTTGATAAGAAGAGTTTGAATGAT AACTCATTGCATTTAAAGATAAATGGCATGCGTTATGAT TTAAAGCTCTTCTTTTTTTATATTTTATAAATTTTGTGAGC AAGCCATCCAATTAATATAAAAAATATCAAATCAAACAC GTTGATATAGATTAAAATTTTAAAAGTTTACATATCAGTT AAGATACAAAATATTCAGACTAATAATTTTCAATTTGGC AAAATATCTTAAACATCAAATTATTATAAGAAATAAATG TATTTAACCATATTCTAGTAATAAAAATATTGAATTTTTA TACTTATTTGTTTAGAATGAACTTTATAACATAGTTGGAT AGAGTTTCGATTTAATAAATTACATGTGAACCTTGCTACA ACAAGATGTGCATCAGAGGAGTGGTTTAATA

Supplementary Table S3. LC-MS/MS results of three promoters

***mecA* promoter**

Accession	Description
Q2FZK7	Bifunctional autolysin OS=Staphylococcus aureus (strain NCTC 8325 / PS 47) OX=93061 GN=atl PE=1 SV=1 UPId=UP000008816 PPId=UP000008816
Q2G2U9	Transcriptional regulator SarA OS=Staphylococcus aureus (strain NCTC 8325 / PS 47) OX=93061 GN=sarA PE=1 SV=3 UPId=UP000008816 PPId=UP000008816
Q2FZS8	Chaperone protein ClpB OS=Staphylococcus aureus (strain NCTC 8325 / PS 47) OX=93061 GN=clpB PE=3 SV=1 UPId=UP000008816 PPId=UP000008816
Q2FZ27	GTP-sensing transcriptional pleiotropic repressor CodY OS=Staphylococcus aureus (strain NCTC 8325 / PS 47) OX=93061 GN=codY PE=1 SV=1 UPId=UP000008816 PPId=UP000008816
A0A380DJ06	Acid phosphatase OS=Staphylococcus aureus OX=1280 GN=hel PE=4 SV=1 UPId=UP000255091 PPId=UP000008816
Q2FXS8	50S ribosomal protein L21 OS=Staphylococcus aureus (strain NCTC 8325 / PS 47) OX=93061 GN=rplU PE=1 SV=1 UPId=UP000008816 PPId=UP000008816
Q2FWW1	MHC class II analog protein OS=Staphylococcus aureus (strain NCTC 8325 / PS 47) OX=93061 GN=SAOUHSC_02161 PE=4 SV=1 UPId=UP000008816 PPId=UP000008816
Q2FXM9	Pyruvate kinase OS=Staphylococcus aureus (strain NCTC 8325 / PS 47) OX=93061 GN=pyk PE=3 SV=1 UPId=UP000008816 PPId=UP000008816
Q2FWE8	ATP synthase subunit alpha OS=Staphylococcus aureus (strain NCTC 8325 / PS 47) OX=93061 GN=atpA PE=3 SV=1 UPId=UP000008816 PPId=UP000008816
Q9F0R1	HTH-type transcriptional regulator SarR OS=Staphylococcus aureus (strain NCTC 8325 / PS 47) OX=93061 GN=sarR PE=1 SV=3 UPId=UP000008816 PPId=UP000008816
A0A380DJX5	Ribose-phosphate pyrophosphokinase OS=Staphylococcus aureus OX=1280 GN=prs PE=3 SV=1 UPId=UP000255091 PPId=UP000008816
Q2G2D0	Translation initiation factor IF-2 OS=Staphylococcus aureus (strain NCTC 8325 / PS 47) OX=93061 GN=infB PE=3 SV=1 UPId=UP000008816 PPId=UP000008816
A0A6B8R4W3	DNA-directed RNA polymerase subunit beta OS=Staphylococcus aureus OX=1280 GN=rpoB PE=3 SV=1 UPId=UP000425526 PPId=UP000008816
Q2G055	Ribosome hibernation promotion factor OS=Staphylococcus aureus (strain NCTC 8325 / PS 47) OX=93061 GN=hpf PE=1 SV=1 UPId=UP000008816 PPId=UP000008816
Q2FW39	30S ribosomal protein S9 OS=Staphylococcus aureus (strain NCTC 8325 / PS 47) OX=93061 GN=rpsI PE=1 SV=1 UPId=UP000008816 PPId=UP000008816
D4FNL7	Ribonuclease R OS=Staphylococcus epidermidis M23864:W2(grey) OX=525375 GN=rnr PE=3 SV=1 UPId=UP000004733 PPId=UP000008816
A0A380EL47	IDP OS=Staphylococcus aureus OX=1280 GN=citC_1 PE=3 SV=1 UPId=UP000254116 PPId=UP000008816
Q2G0P5	ATP-dependent Clp protease ATP-binding subunit ClpC OS=Staphylococcus aureus (strain

	NCTC 8325 / PS 47) OX=93061 GN=clpC PE=1 SV=1 UPId=UP000008816 PPId=UP000008816
Q2FZB8	Fibrinogen-binding protein OS=Staphylococcus aureus (strain NCTC 8325 / PS 47) OX=93061 GN=SAOUHSC_01114 PE=4 SV=1 UPId=UP000008816 PPId=UP000008816
A0A3S0KKL9	Translation initiation factor IF-3 OS=Staphylococcus pasteuri OX=45972 GN=infC PE=3 SV=1 UPId=UP000273693 PPId=UP000008816
Q2G155	Lipase 2 OS=Staphylococcus aureus (strain NCTC 8325 / PS 47) OX=93061 GN=lip2 PE=1 SV=1 UPId=UP000008816 PPId=UP000008816
A0A1D4UZD1	NADH dehydrogenase OS=Staphylococcus aureus OX=1280 GN=ndh PE=4 SV=1 UPId=UP000249913 PPId=UP000008816
A0A2X2KBR6	Iron(III) dicitrate-binding protein OS=Staphylococcus aureus OX=1280 GN=yhfQ_2 PE=4 SV=1 UPId=UP000249913 PPId=UP000008816
Q2FZC7	Succinate dehydrogenase (quinone) OS=Staphylococcus aureus (strain NCTC 8325 / PS 47) OX=93061 GN=SAOUHSC_01105 PE=3 SV=1 UPId=UP000008816 PPId=UP000008816

***sarA* promoter**

Accession	Description
Q2FXM9	Pyruvate kinase OS=Staphylococcus aureus (strain NCTC 8325 / PS 47) OX=93061 GN=pyk PE=3 SV=1 UPId=UP000008816 PPId=UP000008816
Q2G0N0	Elongation factor Tu OS=Staphylococcus aureus (strain NCTC 8325 / PS 47) OX=93061 GN=tuf PE=3 SV=1 UPId=UP000008816 PPId=UP000008816
Q2FYS9	Aconitate hydratase OS=Staphylococcus aureus (strain NCTC 8325 / PS 47) OX=93061 GN=SAOUHSC_01347 PE=3 SV=1 UPId=UP000008816 PPId=UP000008816
Q2G2U9	Transcriptional regulator SarA OS=Staphylococcus aureus (strain NCTC 8325 / PS 47) OX=93061 GN=sarA PE=1 SV=3 UPId=UP000008816 PPId=UP000008816
Q2FZ89	Cell division protein FtsZ OS=Staphylococcus aureus (strain NCTC 8325 / PS 47) OX=93061 GN=ftsZ PE=1 SV=1 UPId=UP000008816 PPId=UP000008816
Q2FZK7	Bifunctional autolysin OS=Staphylococcus aureus (strain NCTC 8325 / PS 47) OX=93061 GN=atl PE=1 SV=1 UPId=UP000008816 PPId=UP000008816
Q2G296	Formate--tetrahydrofolate ligase OS=Staphylococcus aureus (strain NCTC 8325 / PS 47) OX=93061 GN=fhs PE=3 SV=1 UPId=UP000008816 PPId=UP000008816
Q2FZS8	Chaperone protein ClpB OS=Staphylococcus aureus (strain NCTC 8325 / PS 47) OX=93061 GN=clpB PE=3 SV=1 UPId=UP000008816 PPId=UP000008816
Q2FYM1	2-oxoglutarate dehydrogenase E1 component OS=Staphylococcus aureus (strain NCTC 8325 / PS 47) OX=93061 GN=odhA PE=3 SV=1 UPId=UP000008816 PPId=UP000008816
Q2G2A4	Dihydrolipoamide acetyltransferase component of pyruvate dehydrogenase complex OS=Staphylococcus aureus (strain NCTC 8325 / PS 47) OX=93061 GN=SAOUHSC_01042 PE=3 SV=1 UPId=UP000008816 PPId=UP000008816
Q2FYF1	Elastin-binding protein EbpS OS=Staphylococcus aureus (strain NCTC 8325 / PS 47) OX=93061 GN=ebpS PE=1 SV=1 UPId=UP000008816 PPId=UP000008816

O07325	Cell division protein FtsA OS=Staphylococcus aureus (strain NCTC 8325 / PS 47) OX=93061 GN=ftsA PE=1 SV=2 UPId=UP000008816 PPId=UP000008816
Q2G0N1	Elongation factor G OS=Staphylococcus aureus (strain NCTC 8325 / PS 47) OX=93061 GN=fusA PE=1 SV=3 UPId=UP000008816 PPId=UP000008816
Q2G032	Glyceraldehyde-3-phosphate dehydrogenase OS=Staphylococcus aureus (strain NCTC 8325 / PS 47) OX=93061 GN=SAOUHSC_00795 PE=3 SV=1 UPId=UP000008816 PPId=UP000008816
Q2FYU7	Catalase OS=Staphylococcus aureus (strain NCTC 8325 / PS 47) OX=93061 GN=katA PE=2 SV=2 UPId=UP000008816 PPId=UP000008816
Q2G0Y7	Inosine-5'-monophosphate dehydrogenase OS=Staphylococcus aureus (strain NCTC 8325 / PS 47) OX=93061 GN=guaB PE=3 SV=1 UPId=UP000008816 PPId=UP000008816
Q2G1I1	Formate dehydrogenase OS=Staphylococcus aureus (strain NCTC 8325 / PS 47) OX=93061 GN=SAOUHSC_00142 PE=3 SV=1 UPId=UP000008816 PPId=UP000008816
Q2FZJ6	Bifunctional protein FolD OS=Staphylococcus aureus (strain NCTC 8325 / PS 47) OX=93061 GN=folD PE=3 SV=1 UPId=UP000008816 PPId=UP000008816
Q2FV67	1-pyrroline-5-carboxylate dehydrogenase OS=Staphylococcus aureus (strain NCTC 8325 / PS 47) OX=93061 GN=rocA PE=3 SV=1 UPId=UP000008816 PPId=UP000008816
A0A380EHA8	Phosphopyruvate hydratase OS=Staphylococcus aureus OX=1280 GN=eno_1 PE=3 SV=1 UPId=UP000254116 PPId=UP000008816
Q2FW66	Alkaline shock protein 23 OS=Staphylococcus aureus (strain NCTC 8325 / PS 47) OX=93061 GN=asp23 PE=3 SV=1 UPId=UP000008816 PPId=UP000008816
Q2G245	Uncharacterized protein OS=Staphylococcus aureus (strain NCTC 8325 / PS 47) OX=93061 GN=SAOUHSC_01854 PE=4 SV=1 UPId=UP000008816 PPId=UP000008816
A0A2T4Q223	DNA starvation/stationary phase protection protein OS=Staphylococcus warneri OX=1292 GN=BU085_03840 PE=3 SV=1 UPId=UP000240717 PPId=UP000008816
Q2FXZ2	Chaperone protein DnaK OS=Staphylococcus aureus (strain NCTC 8325 / PS 47) OX=93061 GN=dnaK PE=3 SV=1 UPId=UP000008816 PPId=UP000008816
A0A2X2M7W6	ABC transporter substrate-binding protein OS=Staphylococcus aureus OX=1280 GN=psaA_2 PE=3 SV=1 UPId=UP000249913 PPId=UP000008816
Q2FYF9	30S ribosomal protein S1 OS=Staphylococcus aureus (strain NCTC 8325 / PS 47) OX=93061 GN=SAOUHSC_01493 PE=3 SV=1 UPId=UP000008816 PPId=UP000008816
Q2FXK6	30S ribosomal protein S4 OS=Staphylococcus aureus (strain NCTC 8325 / PS 47) OX=93061 GN=rpsD PE=1 SV=1 UPId=UP000008816 PPId=UP000008816
Q2G0Q8	Cysteine synthase OS=Staphylococcus aureus (strain NCTC 8325 / PS 47) OX=93061 GN=SAOUHSC_00488 PE=3 SV=1 UPId=UP000008816 PPId=UP000008816
Q2G1J0	Putative aldehyde dehydrogenase AldA OS=Staphylococcus aureus (strain NCTC 8325 / PS 47) OX=93061 GN=aldA PE=3 SV=1 UPId=UP000008816 PPId=UP000008816
Q2FWB9	Deoxyribose-phosphate aldolase OS=Staphylococcus aureus (strain NCTC 8325 / PS 47) OX=93061 GN=deoC PE=3 SV=1 UPId=UP000008816 PPId=UP000008816

Q2FW11	50S ribosomal protein L22 OS=Staphylococcus aureus (strain NCTC 8325 / PS 47) OX=93061 GN=rplV PE=1 SV=1 UPId=UP000008816 PPId=UP000008816
A0A6H3XPG2	L-lactate dehydrogenase OS=Staphylococcus aureus OX=1280 GN=ldh2_1 PE=4 SV=1 UPId=UP000443692 PPId=UP000008816
Q2G0P5	ATP-dependent Clp protease ATP-binding subunit ClpC OS=Staphylococcus aureus (strain NCTC 8325 / PS 47) OX=93061 GN=clpC PE=1 SV=1 UPId=UP000008816 PPId=UP000008816
Q2FV86	Pyruvate oxidase, putative OS=Staphylococcus aureus (strain NCTC 8325 / PS 47) OX=93061 GN=SAOUHSC_02849 PE=3 SV=1 UPId=UP000008816 PPId=UP000008816
Q2FWE0	Peptide chain release factor 1 OS=Staphylococcus aureus (strain NCTC 8325 / PS 47) OX=93061 GN=prfA PE=3 SV=1 UPId=UP000008816 PPId=UP000008816
Q2FZ92	UDP-N-acetylmuramoylalanine--D-glutamate ligase OS=Staphylococcus aureus (strain NCTC 8325 / PS 47) OX=93061 GN=murD PE=3 SV=1 UPId=UP000008816 PPId=UP000008816
Q2FZH5	Phosphoenolpyruvate-protein phosphotransferase OS=Staphylococcus aureus (strain NCTC 8325 / PS 47) OX=93061 GN=SAOUHSC_01029 PE=3 SV=1 UPId=UP000008816 PPId=UP000008816
Q2G0B5	Uncharacterized protein OS=Staphylococcus aureus (strain NCTC 8325 / PS 47) OX=93061 GN=SAOUHSC_00690 PE=4 SV=1 UPId=UP000008816 PPId=UP000008816
A0A4U0CRW2	MSCRAMM family adhesin clumping factor ClfA (Fragment) OS=Staphylococcus aureus subsp. aureus OX=46170 GN=clfA PE=4 SV=1 UPId=UP000306447 PPId=UP000008816
Q2G170	5'-nucleotidase, lipoprotein e(P4) family OS=Staphylococcus aureus (strain NCTC 8325 / PS 47) OX=93061 GN=SAOUHSC_00284 PE=4 SV=1 UPId=UP000008816 PPId=UP000008816
Q2G0N5	DNA-directed RNA polymerase subunit beta' OS=Staphylococcus aureus (strain NCTC 8325 / PS 47) OX=93061 GN=rpoC PE=3 SV=2 UPId=UP000008816 PPId=UP000008816
Q2FZ82	Isoleucine--tRNA ligase OS=Staphylococcus aureus (strain NCTC 8325 / PS 47) OX=93061 GN=ileS PE=3 SV=1 UPId=UP000008816 PPId=UP000008816
Q2G041	Thioredoxin reductase OS=Staphylococcus aureus (strain NCTC 8325 / PS 47) OX=93061 GN=SAOUHSC_00785 PE=3 SV=1 UPId=UP000008816 PPId=UP000008816
Q2FVT8	Uncharacterized protein OS=Staphylococcus aureus (strain NCTC 8325 / PS 47) OX=93061 GN=SAOUHSC_02604 PE=4 SV=1 UPId=UP000008816 PPId=UP000008816
Q2FWN4	60 kDa chaperonin OS=Staphylococcus aureus (strain NCTC 8325 / PS 47) OX=93061 GN=groL PE=3 SV=1 UPId=UP000008816 PPId=UP000008816
Q2G019	DUF5067 domain-containing protein OS=Staphylococcus aureus (strain NCTC 8325 / PS 47) OX=93061 GN=SAOUHSC_00808 PE=1 SV=1 UPId=UP000008816 PPId=UP000008816
Q2FXP7	Threonine--tRNA ligase OS=Staphylococcus aureus (strain NCTC 8325 / PS 47) OX=93061 GN=thrS PE=3 SV=1 UPId=UP000008816 PPId=UP000008816
Q2FZL5	1,4-dihydroxy-2-naphthoyl-CoA synthase OS=Staphylococcus aureus (strain NCTC 8325 / PS 47) OX=93061 GN=menB PE=3 SV=1 UPId=UP000008816 PPId=UP000008816

Q2FWD6	Putative aldehyde dehydrogenase OS=Staphylococcus aureus (strain NCTC 8325 / PS 47) OX=93061 GN=SAOUHSC_02363 PE=3 SV=1 UPId=UP000008816 PPId=UP000008816
Q2FZ83	Uncharacterized protein OS=Staphylococcus aureus (strain NCTC 8325 / PS 47) OX=93061 GN=SAOUHSC_01158 PE=4 SV=1 UPId=UP000008816 PPId=UP000008816
P47768	DNA-directed RNA polymerase subunit beta OS=Staphylococcus aureus (strain NCTC 8325 / PS 47) OX=93061 GN=rpoB PE=3 SV=2 UPId=UP000008816 PPId=UP000008816
Q2G2D7	PKS_ER domain-containing protein OS=Staphylococcus aureus (strain NCTC 8325 / PS 47) OX=93061 GN=SAOUHSC_02447 PE=4 SV=1 UPId=UP000008816 PPId=UP000008816
Q2G224	Deoxyribose-phosphate aldolase OS=Staphylococcus aureus (strain NCTC 8325 / PS 47) OX=93061 GN=deoC PE=3 SV=1 UPId=UP000008816 PPId=UP000008816
A0A2X2K781	Short-chain alcohol dehydrogenase OS=Staphylococcus aureus OX=1280 GN=NCTC7878_03552 PE=4 SV=1 UPId=UP000249913 PPId=UP000008816
Q2FW20	30S ribosomal protein S8 OS=Staphylococcus aureus (strain NCTC 8325 / PS 47) OX=93061 GN=rpsH PE=1 SV=1 UPId=UP000008816 PPId=UP000008816
Q2G1P6	M20_dimer domain-containing protein OS=Staphylococcus aureus (strain NCTC 8325 / PS 47) OX=93061 GN=SAOUHSC_00057 PE=4 SV=1 UPId=UP000008816 PPId=UP000008816
Q2FY66	Glucose-6-phosphate 1-dehydrogenase OS=Staphylococcus aureus (strain NCTC 8325 / PS 47) OX=93061 GN=zwf PE=3 SV=1 UPId=UP000008816 PPId=UP000008816
Q2G2A5	Pyruvate dehydrogenase complex, E1 component, pyruvate dehydrogenase beta subunit, putative OS=Staphylococcus aureus (strain NCTC 8325 / PS 47) OX=93061 GN=SAOUHSC_01041 PE=4 SV=1 UPId=UP000008816 PPId=UP000008816
Q2FXV9	Alanine--tRNA ligase OS=Staphylococcus aureus (strain NCTC 8325 / PS 47) OX=93061 GN=alaS PE=3 SV=1 UPId=UP000008816 PPId=UP000008816
Q2FW12	30S ribosomal protein S3 OS=Staphylococcus aureus (strain NCTC 8325 / PS 47) OX=93061 GN=rpsC PE=1 SV=1 UPId=UP000008816 PPId=UP000008816
Q2FXE8	Uncharacterized protein OS=Staphylococcus aureus (strain NCTC 8325 / PS 47) OX=93061 GN=SAOUHSC_01901 PE=4 SV=1 UPId=UP000008816 PPId=UP000008816
Q2G0Q3	Lysine--tRNA ligase OS=Staphylococcus aureus (strain NCTC 8325 / PS 47) OX=93061 GN=lysS PE=3 SV=1 UPId=UP000008816 PPId=UP000008816
P48860	50S ribosomal protein L7/L12 OS=Staphylococcus aureus (strain NCTC 8325 / PS 47) OX=93061 GN=rplL PE=3 SV=2 UPId=UP000008816 PPId=UP000008816
Q2G2A3	Dihydrolipoyl dehydrogenase OS=Staphylococcus aureus (strain NCTC 8325 / PS 47) OX=93061 GN=SAOUHSC_01043 PE=3 SV=1 UPId=UP000008816 PPId=UP000008816
Q2FVT5	Urocanate hydratase OS=Staphylococcus aureus (strain NCTC 8325 / PS 47) OX=93061 GN=hutU PE=3 SV=1 UPId=UP000008816 PPId=UP000008816
Q2FW30	30S ribosomal protein S13 OS=Staphylococcus aureus (strain NCTC 8325 / PS 47) OX=93061 GN=rpsM PE=1 SV=1 UPId=UP000008816 PPId=UP000008816
Q2FZ27	GTP-sensing transcriptional pleiotropic repressor CodY OS=Staphylococcus aureus (strain NCTC 8325 / PS 47) OX=93061 GN=codY PE=1 SV=1 UPId=UP000008816

	PPId=UP000008816

***sarR* promoter**

Accession	Description
Q2G245	Uncharacterized protein OS=Staphylococcus aureus (strain NCTC 8325 / PS 47) OX=93061 GN=SAOUHSC_01854 PE=4 SV=1 UPId=UP000008816 PPId=UP000008816
Q2G2U9	Transcriptional regulator SarA OS=Staphylococcus aureus (strain NCTC 8325 / PS 47) OX=93061 GN=sarA PE=1 SV=3 UPId=UP000008816 PPId=UP000008816
Q2FZK7	Bifunctional autolysin OS=Staphylococcus aureus (strain NCTC 8325 / PS 47) OX=93061 GN=atl PE=1 SV=1 UPId=UP000008816 PPId=UP000008816
Q2FZI6	Bifunctional purine biosynthesis protein PurH OS=Staphylococcus aureus (strain NCTC 8325 / PS 47) OX=93061 GN=purH PE=3 SV=1 UPId=UP000008816 PPId=UP000008816
Q2G0N0	Elongation factor Tu OS=Staphylococcus aureus (strain NCTC 8325 / PS 47) OX=93061 GN=tuf PE=3 SV=1 UPId=UP000008816 PPId=UP000008816
Q2FXM9	Pyruvate kinase OS=Staphylococcus aureus (strain NCTC 8325 / PS 47) OX=93061 GN=pyk PE=3 SV=1 UPId=UP000008816 PPId=UP000008816
Q2FXZ2	Chaperone protein DnaK OS=Staphylococcus aureus (strain NCTC 8325 / PS 47) OX=93061 GN=dnaK PE=3 SV=1 UPId=UP000008816 PPId=UP000008816
Q2FYF1	Elastin-binding protein EbpS OS=Staphylococcus aureus (strain NCTC 8325 / PS 47) OX=93061 GN=ebpS PE=1 SV=1 UPId=UP000008816 PPId=UP000008816
Q2FXX0	Acetyl-CoA carboxylase, biotin carboxyl carrier protein, putative OS=Staphylococcus aureus (strain NCTC 8325 / PS 47) OX=93061 GN=SAOUHSC_01710 PE=4 SV=1 UPId=UP000008816 PPId=UP000008816
Q2G1D8	Formate acetyltransferase OS=Staphylococcus aureus (strain NCTC 8325 / PS 47) OX=93061 GN=pflB PE=3 SV=1 UPId=UP000008816 PPId=UP000008816
Q2FW31	30S ribosomal protein S11 OS=Staphylococcus aureus (strain NCTC 8325 / PS 47) OX=93061 GN=rpsK PE=1 SV=1 UPId=UP000008816 PPId=UP000008816
Q2FZ25	30S ribosomal protein S2 OS=Staphylococcus aureus (strain NCTC 8325 / PS 47) OX=93061 GN=rpsB PE=1 SV=2 UPId=UP000008816 PPId=UP000008816
Q9F0R1	HTH-type transcriptional regulator SarR OS=Staphylococcus aureus (strain NCTC 8325 / PS 47) OX=93061 GN=sarR PE=1 SV=3 UPId=UP000008816 PPId=UP000008816
Q2FXM1	Usp domain-containing protein OS=Staphylococcus aureus (strain NCTC 8325 / PS 47) OX=93061 GN=SAOUHSC_01814 PE=3 SV=1 UPId=UP000008816 PPId=UP000008816
Q2G0P0	50S ribosomal protein L1 OS=Staphylococcus aureus (strain NCTC 8325 / PS 47) OX=93061 GN=rplA PE=3 SV=1 UPId=UP000008816 PPId=UP000008816
Q2FXY6	30S ribosomal protein S20 OS=Staphylococcus aureus (strain NCTC 8325 / PS 47) OX=93061 GN=rpsT PE=1 SV=1 UPId=UP000008816 PPId=UP000008816
Q2FW10	30S ribosomal protein S19 OS=Staphylococcus aureus (strain NCTC 8325 / PS 47) OX=93061 GN=rpsS PE=1 SV=1 UPId=UP000008816 PPId=UP000008816
Q2FZJ6	Bifunctional protein FolD OS=Staphylococcus aureus (strain NCTC 8325 / PS 47) OX=93061

	GN=folD PE=3 SV=1 UPId=UP000008816 PPId=UP000008816
Q5HK31	Penicillin-binding protein 2 OS=Staphylococcus epidermidis (strain ATCC 35984 / RP62A) OX=176279 GN=mecA PE=3 SV=1 UPId=UP000000531 PPId=UP000008816
Q2FXH8	Uncharacterized protein OS=Staphylococcus aureus (strain NCTC 8325 / PS 47) OX=93061 GN=SAOUHSC_01869 PE=4 SV=1 UPId=UP000008816 PPId=UP000008816
Q2FZ89	Cell division protein FtsZ OS=Staphylococcus aureus (strain NCTC 8325 / PS 47) OX=93061 GN=ftsZ PE=1 SV=1 UPId=UP000008816 PPId=UP000008816
Q2FWE6	Uracil phosphoribosyltransferase OS=Staphylococcus aureus (strain NCTC 8325 / PS 47) OX=93061 GN=upp PE=3 SV=1 UPId=UP000008816 PPId=UP000008816
Q2G170	5'-nucleotidase, lipoprotein e(P4) family OS=Staphylococcus aureus (strain NCTC 8325 / PS 47) OX=93061 GN=SAOUHSC_00284 PE=4 SV=1 UPId=UP000008816 PPId=UP000008816
Q2G2A4	Dihydrolipoamide acetyltransferase component of pyruvate dehydrogenase complex OS=Staphylococcus aureus (strain NCTC 8325 / PS 47) OX=93061 GN=SAOUHSC_01042 PE=3 SV=1 UPId=UP000008816 PPId=UP000008816
P60430	50S ribosomal protein L2 OS=Staphylococcus aureus (strain NCTC 8325 / PS 47) OX=93061 GN=rplB PE=1 SV=1 UPId=UP000008816 PPId=UP000008816
Q2G0Z2	Uncharacterized protein OS=Staphylococcus aureus (strain NCTC 8325 / PS 47) OX=93061 GN=SAOUHSC_00369 PE=4 SV=1 UPId=UP000008816 PPId=UP000008816
Q2G0B5	Uncharacterized protein OS=Staphylococcus aureus (strain NCTC 8325 / PS 47) OX=93061 GN=SAOUHSC_00690 PE=4 SV=1 UPId=UP000008816 PPId=UP000008816
Q2FXP9	Translation initiation factor IF-3 OS=Staphylococcus aureus (strain NCTC 8325 / PS 47) OX=93061 GN=infC PE=3 SV=1 UPId=UP000008816 PPId=UP000008816
Q2FZJ0	Phosphoribosylformylglycinamidine synthase subunit PurL OS=Staphylococcus aureus (strain NCTC 8325 / PS 47) OX=93061 GN=purL PE=3 SV=1 UPId=UP000008816 PPId=UP000008816
Q2FWB9	Deoxyribose-phosphate aldolase OS=Staphylococcus aureus (strain NCTC 8325 / PS 47) OX=93061 GN=deoC PE=3 SV=1 UPId=UP000008816 PPId=UP000008816
A0A380EK47	General stress protein-like protein OS=Staphylococcus aureus OX=1280 GN=NCTC10702_02729 PE=3 SV=1 UPId=UP000254116 PPId=UP000008816
Q2FZJ3	Phosphoribosylaminoimidazole-succinocarboxamide synthase OS=Staphylococcus aureus (strain NCTC 8325 / PS 47) OX=93061 GN=purC PE=3 SV=1 UPId=UP000008816 PPId=UP000008816
A0A6B0CC41	30S ribosomal protein S7 OS=Staphylococcus aureus OX=1280 GN=rpsG PE=3 SV=1 UPId=UP000432849 PPId=UP000008816
P48860	50S ribosomal protein L7/L12 OS=Staphylococcus aureus (strain NCTC 8325 / PS 47) OX=93061 GN=rplL PE=3 SV=2 UPId=UP000008816 PPId=UP000008816
P02976	Immunoglobulin G-binding protein A OS=Staphylococcus aureus (strain NCTC 8325 / PS 47) OX=93061 GN=spa PE=1 SV=3 UPId=UP000008816 PPId=UP000008816
Q2FWN4	60 kDa chaperonin OS=Staphylococcus aureus (strain NCTC 8325 / PS 47) OX=93061

	GN=groL PE=3 SV=1 UPId=UP000008816 PPId=UP000008816
Q2FYF9	30S ribosomal protein S1 OS=Staphylococcus aureus (strain NCTC 8325 / PS 47) OX=93061 GN=SAOUHSC_01493 PE=3 SV=1 UPId=UP000008816 PPId=UP000008816
Q2FW12	30S ribosomal protein S3 OS=Staphylococcus aureus (strain NCTC 8325 / PS 47) OX=93061 GN=rpsC PE=1 SV=1 UPId=UP000008816 PPId=UP000008816
A0A6H5DTD2	DNA-directed RNA polymerase OS=Staphylococcus aureus OX=1280 GN=rpoB PE=4 SV=1 UPId=UP000445221 PPId=UP000008816
A0A2X2M7W6	ABC transporter substrate-binding protein OS=Staphylococcus aureus OX=1280 GN=psaA_2 PE=3 SV=1 UPId=UP000249913 PPId=UP000008816
Q2G296	Formate--tetrahydrofolate ligase OS=Staphylococcus aureus (strain NCTC 8325 / PS 47) OX=93061 GN=fhs PE=3 SV=1 UPId=UP000008816 PPId=UP000008816
Q2FYU7	Catalase OS=Staphylococcus aureus (strain NCTC 8325 / PS 47) OX=93061 GN=katA PE=2 SV=2 UPId=UP000008816 PPId=UP000008816
Q2FZT4	Uncharacterized protein SAOUHSC_00906 OS=Staphylococcus aureus (strain NCTC 8325 / PS 47) OX=93061 GN=SAOUHSC_00906 PE=3 SV=1 UPId=UP000008816 PPId=UP000008816
Q2FW20	30S ribosomal protein S8 OS=Staphylococcus aureus (strain NCTC 8325 / PS 47) OX=93061 GN=rpsH PE=1 SV=1 UPId=UP000008816 PPId=UP000008816
Q2FVN6	Uncharacterized protein OS=Staphylococcus aureus (strain NCTC 8325 / PS 47) OX=93061 GN=SAOUHSC_02666 PE=4 SV=1 UPId=UP000008816 PPId=UP000008816
Q2G0B1	HTH-type transcriptional regulator MgrA OS=Staphylococcus aureus (strain NCTC 8325 / PS 47) OX=93061 GN=mgrA PE=1 SV=3 UPId=UP000008816 PPId=UP000008816
Q2G028	Enolase OS=Staphylococcus aureus (strain NCTC 8325 / PS 47) OX=93061 GN=eno PE=1 SV=1 UPId=UP000008816 PPId=UP000008816
Q2G1G5	PTS system EIIBC component SAOUHSC_00158 OS=Staphylococcus aureus (strain NCTC 8325 / PS 47) OX=93061 GN=SAOUHSC_00158 PE=3 SV=1 UPId=UP000008816 PPId=UP000008816
A0A431ZSC5	Cyclic pyranopterin monophosphate synthase OS=Staphylococcus pasteuri OX=45972 GN=moaC PE=3 SV=1 UPId=UP000273693 PPId=UP000008816
Q2G2D8	ABC transporter, substrate-binding protein, putative OS=Staphylococcus aureus (strain NCTC 8325 / PS 47) OX=93061 GN=SAOUHSC_00634 PE=3 SV=1 UPId=UP000008816 PPId=UP000008816
A0A431ZM32	Endolytic murein transglycosylase OS=Staphylococcus pasteuri OX=45972 GN=mltG PE=3 SV=1 UPId=UP000273693 PPId=UP000008816
Q2FY10	Putative pyruvate, phosphate dikinase regulatory protein OS=Staphylococcus aureus (strain NCTC 8325 / PS 47) OX=93061 GN=SAOUHSC_01664 PE=3 SV=1 UPId=UP000008816 PPId=UP000008816
Q2G224	Deoxyribose-phosphate aldolase OS=Staphylococcus aureus (strain NCTC 8325 / PS 47) OX=93061 GN=deoC PE=3 SV=1 UPId=UP000008816 PPId=UP000008816

Q2FXZ7	30S ribosomal protein S21 OS=Staphylococcus aureus (strain NCTC 8325 / PS 47) OX=93061 GN=rpsU PE=1 SV=1 UPId=UP000008816 PPId=UP000008816
Q2G2G0	DM13 domain-containing protein OS=Staphylococcus aureus (strain NCTC 8325 / PS 47) OX=93061 GN=SAOUHSC_00717 PE=4 SV=1 UPId=UP000008816 PPId=UP000008816
A0A2X2M757	50S ribosomal protein L6 OS=Staphylococcus aureus OX=1280 GN=rplF_1 PE=4 SV=1 UPId=UP000249913 PPId=UP000008816
Q2G0Y7	Inosine-5'-monophosphate dehydrogenase OS=Staphylococcus aureus (strain NCTC 8325 / PS 47) OX=93061 GN=guaB PE=3 SV=1 UPId=UP000008816 PPId=UP000008816
A0A380EDA2	Polyribitolphosphotransferase OS=Staphylococcus aureus OX=1280 GN=tagF_1 PE=3 SV=1 UPId=UP000254116 PPId=UP000008816
Q2FWN9	Uncharacterized leukocidin-like protein 2 OS=Staphylococcus aureus (strain NCTC 8325 / PS 47) OX=93061 GN=SAOUHSC_02243 PE=1 SV=1 UPId=UP000008816 PPId=UP000008816
Q2FWX9	4,4'-diaponeurosporen-aldehyde dehydrogenase OS=Staphylococcus aureus (strain NCTC 8325 / PS 47) OX=93061 GN=aldH1 PE=1 SV=1 UPId=UP000008816 PPId=UP000008816
Q2FZ23	Elongation factor Ts OS=Staphylococcus aureus (strain NCTC 8325 / PS 47) OX=93061 GN=tsf PE=3 SV=1 UPId=UP000008816 PPId=UP000008816
A0A2T4PZP2	Cysteine synthase OS=Staphylococcus warneri OX=1292 GN=cysK PE=3 SV=1 UPId=UP000240717 PPId=UP000008816
Q2FV67	1-pyrroline-5-carboxylate dehydrogenase OS=Staphylococcus aureus (strain NCTC 8325 / PS 47) OX=93061 GN=rocA PE=3 SV=1 UPId=UP000008816 PPId=UP000008816
A0A2T4PYT6	Glutamate ligase OS=Staphylococcus warneri OX=1292 GN=BU085_09705 PE=4 SV=1 UPId=UP000240717 PPId=UP000008816
Q2FZC2	Fibrinogen-binding protein OS=Staphylococcus aureus (strain NCTC 8325 / PS 47) OX=93061 GN=SAOUHSC_01110 PE=4 SV=1 UPId=UP000008816 PPId=UP000008816
Q2G0F2	Uncharacterized protein OS=Staphylococcus aureus (strain NCTC 8325 / PS 47) OX=93061 GN=SAOUHSC_00617 PE=4 SV=1 UPId=UP000008816 PPId=UP000008816
Q2FW30	30S ribosomal protein S13 OS=Staphylococcus aureus (strain NCTC 8325 / PS 47) OX=93061 GN=rpsM PE=1 SV=1 UPId=UP000008816 PPId=UP000008816
Q2FZ07	Uncharacterized protein OS=Staphylococcus aureus (strain NCTC 8325 / PS 47) OX=93061 GN=SAOUHSC_01264 PE=4 SV=1 UPId=UP000008816 PPId=UP000008816
Q2FW08	50S ribosomal protein L23 OS=Staphylococcus aureus (strain NCTC 8325 / PS 47) OX=93061 GN=rplW PE=1 SV=1 UPId=UP000008816 PPId=UP000008816
A0A380DXF4	Pyruvate oxidase OS=Staphylococcus aureus OX=1280 GN=poxB PE=3 SV=1 UPId=UP000255091 PPId=UP000008816
Q2FZV7	NADH dehydrogenase-like protein SAOUHSC_00878 OS=Staphylococcus aureus (strain NCTC 8325 / PS 47) OX=93061 GN=SAOUHSC_00878 PE=1 SV=1 UPId=UP000008816 PPId=UP000008816
Q2FZJ9	Probable quinol oxidase subunit 2 OS=Staphylococcus aureus (strain NCTC 8325 / PS 47)

	OX=93061 GN=qoxA PE=3 SV=1 UPId=UP000008816 PPId=UP000008816
Q2FXQ6	Trigger factor OS=Staphylococcus aureus (strain NCTC 8325 / PS 47) OX=93061 GN=tig PE=3 SV=1 UPId=UP000008816 PPId=UP000008816
Q5HNZ8	50S ribosomal protein L33 1 OS=Staphylococcus epidermidis (strain ATCC 35984 / RP62A) OX=176279 GN=rpmG1 PE=3 SV=1 UPId=UP000000531 PPId=UP000008816
Q2G273	Urease accessory protein UreG OS=Staphylococcus aureus (strain NCTC 8325 / PS 47) OX=93061 GN=ureG PE=3 SV=1 UPId=UP000008816 PPId=UP000008816
Q2FWW1	MHC class II analog protein OS=Staphylococcus aureus (strain NCTC 8325 / PS 47) OX=93061 GN=SAOUHSC_02161 PE=4 SV=1 UPId=UP000008816 PPId=UP000008816
Q2FY42	Biotin carboxyl carrier protein of acetyl-CoA carboxylase OS=Staphylococcus aureus (strain NCTC 8325 / PS 47) OX=93061 GN=SAOUHSC_01624 PE=4 SV=1 UPId=UP000008816 PPId=UP000008816
Q2FZ45	30S ribosomal protein S16 OS=Staphylococcus aureus (strain NCTC 8325 / PS 47) OX=93061 GN=rpsP PE=1 SV=1 UPId=UP000008816 PPId=UP000008816
Q2G1U6	Regulatory protein Spx OS=Staphylococcus aureus (strain NCTC 8325 / PS 47) OX=93061 GN=spxA PE=3 SV=1 UPId=UP000008816 PPId=UP000008816
Q2FV17	Fructose-bisphosphate aldolase class 1 OS=Staphylococcus aureus (strain NCTC 8325 / PS 47) OX=93061 GN=fda PE=3 SV=1 UPId=UP000008816 PPId=UP000008816
Q2FZP9	Putative phosphoesterase SAOUHSC_00951 OS=Staphylococcus aureus (strain NCTC 8325 / PS 47) OX=93061 GN=SAOUHSC_00951 PE=3 SV=1 UPId=UP000008816 PPId=UP000008816
Q2G111	30S ribosomal protein S18 OS=Staphylococcus aureus (strain NCTC 8325 / PS 47) OX=93061 GN=rpsR PE=1 SV=1 UPId=UP000008816 PPId=UP000008816
Q2G0Z0	YflT domain-containing protein OS=Staphylococcus aureus (strain NCTC 8325 / PS 47) OX=93061 GN=SAOUHSC_00371 PE=3 SV=1 UPId=UP000008816 PPId=UP000008816
Q2FW07	50S ribosomal protein L4 OS=Staphylococcus aureus (strain NCTC 8325 / PS 47) OX=93061 GN=rplD PE=1 SV=1 UPId=UP000008816 PPId=UP000008816
A0A6H3W178	Elongation factor G OS=Staphylococcus aureus OX=1280 GN=fusA_2 PE=3 SV=1 UPId=UP000444022 PPId=UP000008816
Q2FX90	Uncharacterized protein OS=Staphylococcus aureus (strain NCTC 8325 / PS 47) OX=93061 GN=SAOUHSC_01987 PE=4 SV=1 UPId=UP000008816 PPId=UP000008816
Q2FWZ8	Bacterial non-heme ferritin OS=Staphylococcus aureus (strain NCTC 8325 / PS 47) OX=93061 GN=ftnA PE=1 SV=1 UPId=UP000008816 PPId=UP000008816
Q2G2G6	DUF4064 domain-containing protein OS=Staphylococcus aureus (strain NCTC 8325 / PS 47) OX=93061 GN=SAOUHSC_01050 PE=4 SV=1 UPId=UP000008816 PPId=UP000008816
Q2FY06	GTPase Era OS=Staphylococcus aureus (strain NCTC 8325 / PS 47) OX=93061 GN=era PE=3 SV=2 UPId=UP000008816 PPId=UP000008816
Q2FYG7	Nucleoside diphosphate kinase OS=Staphylococcus aureus (strain NCTC 8325 / PS 47) OX=93061 GN=ndk PE=3 SV=2 UPId=UP000008816 PPId=UP000008816

A0A2X2K2P2	HTH-type transcriptional regulator sarS OS=Staphylococcus aureus OX=1280 GN=sarS_2 PE=4 SV=1 UPId=UP000249913 PPId=UP000008816

Supplementary Table S4. Sensititre data

**Antibiotics**	**USA300 JE2 MIC (μg/ml)**	**NE1193 *ΔsarA* (μg/ml)**
Daptomycin	≤0.5	≤0.5
Oxacillin + 2% NaCl	≥4	≥4
Ampicillin	8	2
Penicillin	8	1
Vancomycin	1	1

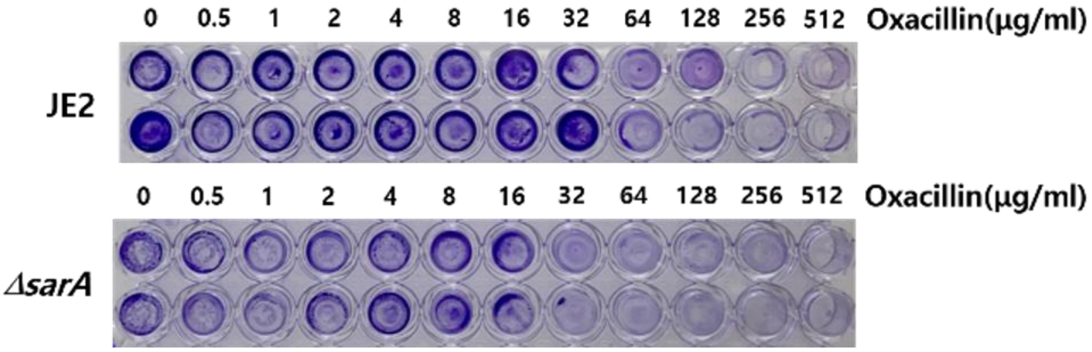

Supplementary Figure S1. Comparison of biofilm formation through crystal violet staining in 96-well plate.
